# The impact of educational environment on academic thriving among medical students: insights from a multinational cross-sectional survey

**DOI:** 10.1186/s12909-025-08053-2

**Published:** 2025-10-21

**Authors:** Zainalabideen Yasser Jumaa, Alaa Hamza Hermis, Ahmed Dahshan, Saleh Zaghir Mohammed Alhetar, Nadia Mohamed Ibrahim Wahba, Fatima Adnan Hallaj, Zeyad Yassin, Ali Jassim Mohammed, Haider Abd AL Ameer Twair, Sameer A. Alkubati, Marwa Ibrahim Mahfouz Khalil, Tarek M. Selim

**Affiliations:** 1 Community Health Nursing, College of Nursing, University of Al Qadisiyah, Al Dewaynia, Iraq; 2 College of Nursing, University of Al Qadisiyah, Al Dewaynia, Iraq; 3 Neurology Department, Cairo University, Cairo, Egypt; 4 Maternal and Child Health Nursing, College of Nursing, University of Ha’il, Hail, Saudi Arabia; 5 Obstetrics and Gynecologic Nursing, Nursing Department, Faculty of Medicine and Health Sciences, Taiz University, Taiz, Yemen; 6 College of Nursing, Prince Sattam Bin Abdulaziz University, Al-Kharj, Saudi Arabia; 7 Psychiatric Nursing and Mental Health, Faculty of Nursing, Port Said University, Port Said, Egypt; 8 Community and Family Medicine, Faculty of Medicine, Latakia University, Lattakia, Syria; 9 Faculty of Medicine, Al Azhar University, Cairo, Egypt; 10 College of nursing, Al-Mustaqbal University, Babil, Iraq; 11 Medical-Surgical Nursing, College of Nursing, University of Hail, Hail, Saudi Arabia; 12 Department of Nursing, Faculty of Medicine and Health Sciences, Hodeida University, Hodeida, Yemen; 13 Gerontological Nursing, Faculty of Nursing, Alexandria University, Alexandria, Egypt; 14 Gerontological Nursing, Faculty of Nursing, Mansoura University, Mansoura, Egypt; 15 Faculty of Applied Medical Sciences, Prince Sattam Bin Abdulaziz University, Al-Kharj, Saudi Arabia

**Keywords:** Medical education, DREEM, Thriving quotient, Arabic countries, Student perceptions, Educational environment, Academic thriving

## Abstract

**Background:**

Academic thriving encompasses students’ cognitive engagement, emotional well-being, and sense of belonging. The educational environment plays a vital role in supporting thriving, particularly in the demanding context of medical education. Limited multinational data exists on how educational environments influence thriving in MENA region. This study was designed to investigate the relationship between the educational environment and academic thriving among medical students across Arabic-speaking countries, using validated assessment tools.

**Methods:**

A cross-sectional, descriptive-correlational design was employed. A total of 1,246 undergraduate medical students from five Arab countries participated in an online survey conducted between February and March 2025. The Dundee Ready Education Environment Measure (DREEM) assessed perceptions of the educational environment, while the Thriving Quotient (TQ) evaluated students’ academic engagement and well-being. Data were analyzed using descriptive statistics, inferential tests, correlation, and regression analyses.

**Results:**

Participants reported generally positive perceptions of their educational environment (mean DREEM score: 113.79 ± 27.76) and moderate levels of thriving (Overall TQ mean was 95.38 ± 18.22). Strongest correlations with academic thriving were found in the domains of academic self-perception and social self-perception. Regression analysis revealed that educational environment variables explained 38.7% of the variance in thriving outcomes (*p* < 0.001). Socio-demographic variables, including gender, financial status, and awareness of student support services, significantly influenced both DREEM and TQ scores.

**Conclusions:**

The educational environment plays a significant role in shaping academic thriving among medical students. Institutions should focus on improving academic support, fostering inclusive environments, and strengthening student-centered teaching strategies to enhance both learning and psychological outcomes.

**Clinical trial number:**

Not applicable.

**Supplementary Information:**

The online version contains supplementary material available at 10.1186/s12909-025-08053-2.

## Introduction

The educational environment plays a pivotal role in shaping students’ academic experiences, well-being, and professional development, particularly in health professions such as medicine and nursing [1]. A supportive and stimulating learning climate enhances motivation, engagement, and ultimately, student success [2]. However, negative or poorly structured environments can lead to stress, burnout, and reduced academic performance, especially among medical students who face high academic and emotional demands [3]. Academic thriving, defined as the ability of students to grow both personally and academically despite challenges, has emerged as an important construct in understanding student resilience and success [4]. Thriving encompasses not only academic achievement but also psychological and social well-being, making it a holistic indicator of student progress [5]. Several factors have been identified as influencing academic thriving, including institutional support services, curriculum design, peer relationships, and financial stability [6]. The Dundee Ready Educational Environment Measure (DREEM) is a widely used tool to assess the quality of educational environments across various medical and health sciences institutions globally [7]. It provides a comprehensive evaluation of five key domains: students’ perceptions of learning, teachers, academic self-perception, atmosphere, and social self-perception [8]. Similarly, the Thriving Quotient (TQ) offers insights into how students perceive their academic and personal growth within these environments. Despite the global use of these tools, limited research has explored the relationship between educational environmental quality and academic thriving specifically in Arabic-speaking countries [9]. Cultural, economic, and institutional differences may influence how students perceive their learning environments and experience academic growth. The impact of the educational environment on academic thriving among medical students can be effectively framed through the integration of Self-Determination Theory (SDT) and Social Constructivism. Self-Determination Theory, developed by Deci and Ryan, posits that individuals possess basic psychological needs for autonomy, competence, and relatedness. When these needs are satisfied, individuals are more likely to experience intrinsic motivation, engagement, and overall well-being. In the context of medical education, a supportive educational environment that fosters autonomy (such as opportunities for self-directed learning), competence (through constructive feedback), and relatedness (via supportive peer and faculty relationships) can significantly enhance students’ academic thriving. This theory aligns well with the dimensions of the DREEM instrument, which can be used to assess how well these psychological needs are met within the educational context (Shen et al., 2024; Kritikou & Giovazolias, 2022).

On the other hand, Social Constructivism, rooted in the works of Vygotsky, emphasizes the role of social interactions and cultural context in learning, suggesting that knowledge is constructed through collaborative experiences and social engagement. This perspective highlights the importance of the educational atmosphere and peer interactions, which shape students’ perceptions and experiences. By assessing dimensions such as Students’ Perception of Atmosphere and Social Self-Perception, the study can explore how collaborative learning environments contribute to academic thriving among medical students. The integration of these two frameworks provides a comprehensive understanding of how the educational environment influences academic thriving; while SDT focuses on the individual psychological needs that drive motivation and engagement, Social Constructivism emphasizes the critical role of social context and interaction in the learning process. Together, these theories enable a deeper exploration of the factors that contribute to medical students’ success across diverse educational settings like those found in the Arab world and MENA countries (Do et al., 2023). This study aims to explore the multifaceted interactions between the educational environment and academic thriving among medical students across various countries, highlighting both commonalities and unique challenges faced by students in different settings. Through a comprehensive multinational survey, this research seeks to identify salient themes and patterns that emerge from students’ experiences.

## Methods

### Study design, setting and participants

This study employed a cross-sectional descriptive-correlational design to explore the relationship between the educational environment and academic thriving among medical students across multiple institutions in Arabic-speaking countries. The study was conducted between February and March 2025 and included undergraduate medical students from five Arab countries: Egypt, Iraq, Saudi Arabia, Yemen, and Syria. Participants were selected using a stratified sampling method to ensure representation from various educational contexts and cultural backgrounds. Participants were selected using a stratified sampling method to ensure a comprehensive representation of medical students from five Arabic-speaking countries: Egypt, Iraq, Saudi Arabia, Yemen, and Syria. Stratification was based on three dimensions: country, year level, and faculty type. This approach allowed for a nuanced exploration of how educational environments and cultural factors vary across these nations. Participants were stratified by year level, encompassing students from the 1 st to the 6th year, capturing a wide spectrum of experiences that influence academic thriving at different stages of medical education. Additionally, stratification by faculty type included Nursing, Medicine, Pharmacy, Dentistry, Technical Health, and Physical Therapy, enabling comparisons across disciplines regarding their educational impacts. This rigorous stratification method ensures a robust and diverse sample, enhancing the generalizability of findings and providing valuable insights into the factors affecting academic thriving in the region.

Inclusion criteria included currently enrolled as a full-time undergraduate medical student, willingness to participate voluntarily and proficiency in reading and understanding Arabic or English. Responses that were incomplete, as well as those submitted by non-medical students who unintentionally accessed the survey, were excluded. A total of 1,246 valid responses were collected and included in the final analysis. To ensure robust data collection, we implemented a strategic recruitment process directed at medical students across multiple accredited medical schools. Participants were invited via institutional emails and relevant online platforms, enhancing the visibility of the study. To foster participation, we provided a clear explanation of the study’s objectives and maintained strict confidentiality regarding responses. We estimated a response rate between 30 and 50%, in line with comparable research. The actual response rate for the study was 40.5%, determined from the total number of medical students invited (3075) and the number of completed surveys received (1246). This response rate reflects a successful recruitment strategy and strong participant engagement, aligning with findings from similar studies in the field. The structured questionnaire was accessible through a user-friendly Google Form, allowing participants to complete the survey at their convenience, thereby optimizing engagement and response rates.

This study received ethical approval from the Research Ethics Committee at the Faculty of Nursing, Al Qadisiyah University in Iraq (Nur 6/2025, Date: 3/3/2025). Following this approval, formal requests were sent to the Vice Dean for Education and Student Affairs at various medical schools in different countries, seeking permission to conduct the research. Administrators at the participating institutions were informed about the study’s purpose before obtaining written consent from the relevant authorities.

Each participant received a detailed explanation of the study’s objectives and was required to provide digital informed consent prior to data collection. This communication emphasized anonymity, voluntary participation, and the right to withdraw at any time. Measures were implemented to ensure data confidentiality and safeguard participants’ privacy. An introductory paragraph outlining the study’s goals, along with assurances of anonymity and confidentiality, was presented before enrollment. Participants were invited to provide digital consent and complete the online form only if they met the inclusion criteria. Those who opted not to participate received a message of appreciation, and their responses were not collected. All survey data were gathered anonymously, with no identifying information recorded, and access was restricted to the principal investigator. The research adhered to the ethical principles set forth in the most recent version of the Declaration of Helsinki, revised in Fortaleza in 2013 by the World Medical Association (WMA).

## Instruments and data collection

Data were collected using a self-administered online questionnaire that included 3 main sections:

- Demographic Data including student demographic characteristics, academic status, health, social, psychological, and economic characteristics.
- Educational Environment: The Arabic version of the Dundee Ready Education Environment Measure (DREEM) was used to assess students’ perceptions of their educational environment. It contains 50 items covering five domains: students’ perceptions of learning (SPL), perceptions of teachers (SPT), academic self-perception (SAP), perceptions of atmosphere (SPA), and social self-perception (SSP). Responses are scored on a 5-point Likert scale ranging from 0 (“strongly disagree”) to 4 (“strongly agree”). Negatively worded items are reverse scored. The maximum global score is 200, with higher scores indicating a more positive perception [ 10].
- Academic Thriving: it was measured using the Thriving Quotient (TQ), adapted and validated for academic use. It is a 21-item tool measuring academic thriving across five dimensions; academic self-efficacy, social integration, emotional regulation, engagement and resilience. Items are rated on a 5-point Likert scale, with total scores ranging from 21 to 105. Higher scores reflect higher levels of academic thriving [ 11, 12].

The survey was distributed via Google Forms. An introductory page provided detailed information about the study’s purpose, confidentiality, voluntary participation, and the right to withdraw. Digital informed consent was obtained before accessing the questionnaire. For full transparency, an English version of the complete questionnaire is provided as Supplementary File 1.

### Statistical analysis

Collected data were analyzed using IBM SPSS version 26.0. Descriptive statistics were used to summarize demographic characteristics and mean scores of DREEM and TQ. Cronbach’s alpha was computed to assess the internal consistency of the instruments, ensuring their reliability. Inferential statistics included independent samples t-tests and one-way ANOVA to compare scores across subgroups. Pearson correlation coefficients were calculated to examine relationships between DREEM and TQ scores. Multiple linear regression analysis was conducted to identify predictors of academic thriving, with statistical significance set at *p* < 0.05. Assumption testing was also performed, including checks for normality and multicollinearity, to validate the appropriateness of our statistical analyses. The results indicated that the majority of our data met the normality assumption, allowing us to proceed with parametric analyses. Our analysis showed that all VIF values were below the acceptable threshold, confirming that multicollinearity was not an issue in our regression model.

## Results

### Demographic characteristics

A total of 1,246 medical students participated in the study. The sample included students from five Arabic-speaking countries: Iraq, Egypt, Syria, Yemen, and Saudi Arabia. Of the participants, 53.6% were females, and the majority (78.9%) were aged between 18 and 23 years. Over half of the respondents (58.4%) reported a “fair” monthly family income, while 23.1% indicated a “good” income and 18.5% a “poor” income. Most students (72.3%) lived in urban areas, and nearly one-third (31.7%) were employed part-time during their studies. Table 1 shows the sociodemographic characteristics of the participants.

**Table 1:** Socio-Demographic characteristics of the study participants (*n* = 1246)

Socio-demographic characteristics	No.	%
Age (Yeas)		
< 17	23	1.8
17–21	705	56.6
> 21	518	41.6
Mean ± SD	21.7 ± 6.13	
Sex		
Male	529	42.5
Female	717	57.5
Residence		
Urban	926	74.3
Rural	320	25.7
Countries		
Egypt	250	20.1
Iraq	238	19.1
Saudi Arabia	243	19.5
Yemen	253	20.3
Syria	262	21.0
Income		
Good	470	37.7
Fair	700	56.2
Poor	76	6.1
Working during studying		
Not working	847	68.0
Working and affecting my study	175	14.0
Working and not affecting my study	224	18.0
Main reasons for working		
Not applicable	847	68.0
Earn more money	246	19.7
More experience for my carrier	153	12.3
Financial support for students		
I don’t know	477	38.3
No	468	37.6
Yes	301	24.2
Faculty		
Nursing	573	46.0
Medicine	394	31.6
Pharmacy	110	8.8
Dentistry	74	5.9
Technical health	49	3.9
Physical therapy	46	3.7
Academic year		
1st	143	11.5
2nd	200	16.1
3rd	347	27.8
4th	266	21.3
5th	108	8.7
6th	182	14.6
Academic achievement		
Not trapped	1063	85.3
Trapped	183	14.7
Any support for trapped students		
Yes	717	57.5
No	529	42.5
Lives with		
With Family	928	74.5
Without family	318	25.5
Support center for students		
I don’t know	649	52.1
No	347	27.8
Yes	250	20.1
Medical condition		
Suffering health problems	324	26.0
Not Suffering health problems	922	74.0
Regular medication		
Yes	289	23.2
No	957	76.8
Suffering critical condition need urgent aid		
Yes	136	10.9
No	1110	89.1
Health Support center for students		
I don’t know	454	36.4
No	312	25.0
Yes	480	38.5
Psychological condition		
Suffering Psychological problems	351	28.2
Not Suffering Psychological problems	895	71.8
Psychiatric Support center for students		
I don’t know	493	39.6
No	468	37.6
Yes	285	22.9
Hobbies		
No	909	73.0
Yes	337	27.0
Involving with student activities		
Yes	737	59.1
No	509	40.9
Student activities Support center		
I don’t know	468	37.6
No	197	15.8
Yes	581	46.6

### Perceptions of the educational environment

The overall mean DREEM score was 113.79 ± 27.76, indicating that students perceived the educational environment as more positive than negative. Table 2 presents detailed domain-specific scores.

**Table 2:** DREEM scores reflecting medical students’ perceptions of Learning, Teaching, and atmosphere (*n* = 1246)

DREEM	Very poor		Plenty of problems		More positive than negative		Excellent		Total score		Mean percent score	
	No.	%	No.	%	No.	%	No.	%	Mean	±SD	Mean	±SD
Students’ Perception of Learning (SpoL)	47	3.8	443	35.6	663	53.2	93	7.5	26.40	7.46	55.01	15.54
Students’ Perception of Teaching (SpoT)	18	1.4	362	29.1	708	56.8	158	12.7	26.12	6.62	59.36	15.04
Students’ Academic Self-Perception (SASP)	56	4.5	299	24.0	673	54.0	218	17.5	19.87	6.26	62.08	19.56
Students’ Perception of the Atmosphere (SpoA)	68	5.5	429	34.4	647	51.9	102	8.2	26.34	7.94	54.87	16.54
Students’ Social Self-Perception (SSSP)	82	6.6	467	37.5	614	49.3	83	6.7	15.06	4.70	53.79	16.79
Overall DREEM	**27**	**2.2**	**371**	**29.8**	**741**	**59.5**	**107**	**8.6**	**113.79**	**27.76**	**56.89**	**13.88**

## Academic thriving scores

The overall mean TQ score was 95.38 ± 18.22, indicating moderate levels of academic thriving among participants. Domain-specific TQ scores are summarized in Table 3.

**Table 3:** TQ scores indicating dimensions of medical student engagement and determination (*n* = 1246)

TQ	Total score		Mean percent score	
	Mean	±SD	Mean	±SD
Engaged Learning	15.22	4.74	56.12	23.69
Academic Determination	27.43	5.65	58.37	16.13
Diverse Citizenship	24.31	6.37	61.02	21.23
Positive Perspective	7.01	0.96	50.07	9.64
Social Connectedness	21.42	5.86	51.39	19.54
Overall TQ	95.38	18.22	55.39	13.02

These findings suggest that while students demonstrate motivation and engagement, there may be limitations in their sense of social belonging and broader perspective.

Table 3 presents the TQ scores reflecting dimensions of medical student engagement and determination among 1,246 participants. The results indicate varying levels of engagement across different dimensions. Notably, Diverse Citizenship scored the highest mean percent (61.02%), suggesting that students feel a strong sense of belonging and responsibility within diverse communities. In contrast, the Positive Perspective dimension had the lowest mean percent score (50.07%), indicating potential areas for improvement in fostering a more optimistic outlook among students. Overall, the Total TQ score of 95.38 (55.39% mean percent) highlights moderate engagement levels, suggesting that while students exhibit significant academic determination and engagement, there remains room for enhancing their social connectedness and positive perspectives to further support their academic success.

## Relationship between educational environment and thriving

**Fig. 1:**
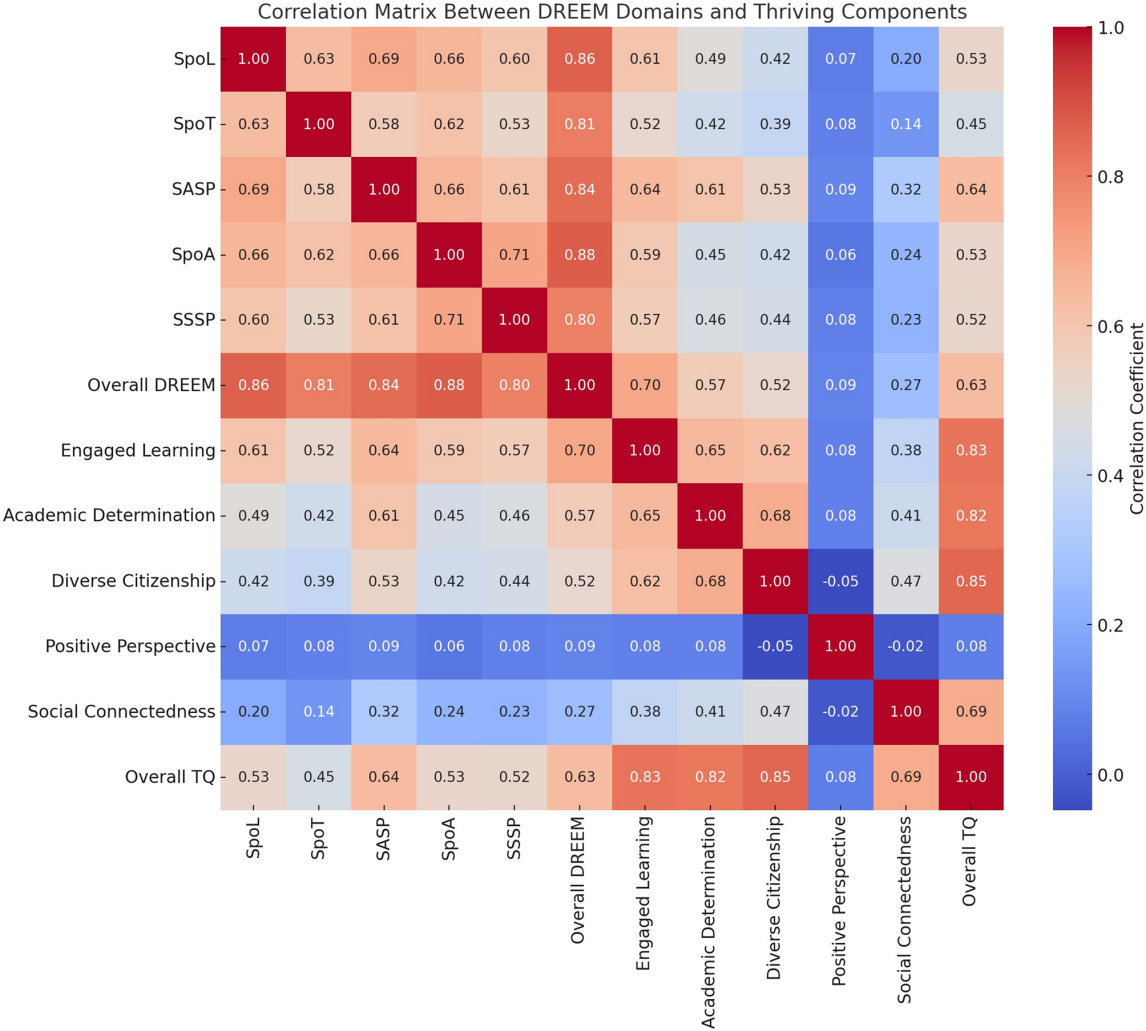
Heatmap visualizing the relationships between DREEM subdomains and components of the Thriving Quotient. Stronger positive correlations appear in warmer colors (reds), and weaker or negative correlations appear in cooler colors (blues)

Pearson’s correlation showed significant positive correlations between the total DREEM score and the overall thriving score (*r* = 0.57, *p* < 0.001). Among the DREEM domains, academic self-perception (*r* = 0.61, *p* < 0.001) and social self-perception (*r* = 0.48, *p* < 0.001) had the strongest associations with thriving, Fig. 1.

### Influence of demographic factors

Female students reported significantly higher thriving scores compared to males (t = 3.14, *p* = 0.002). Students from clinical years also had significantly higher DREEM and thriving scores than those in preclinical years (*p* < 0.05). Awareness of support services plays a role as students from urban areas had a higher mean DREEM score (114.47) compared to their rural counterparts (111.81). Income category showed significant differences across groups, as students with “Good” income had the highest mean scores on both DREEM (116.40) and TQ (96.70).

The data reveals significant differences in the educational environment and thriving across several Middle Eastern countries. Saudi Arabia stands out with the highest DREEM score of 126.79, suggesting an optimal educational environment, followed by Iraq at 119.34 and Yemen at 117.96. In contrast, Egypt and Syria exhibit the lowest scores, with Egypt at 97.04 and Syria at 108.96, indicating challenges in their educational systems. For teaching quality, Saudi Arabia again leads with a mean of 102.52, while Egypt’s score of 90.29 reflects the most critical perception of teaching quality. Notably, Yemen’s DREEM and TQ scores show significant statistical differences (F = 27.746, *p* < 0.001 for DREEM and F = 10.389, *p* < 0.001 for TQ), highlighting substantial variations that warrant further investigation. Overall, the findings underscore the need for targeted improvements in Egypt and Syria, while exploring the successful strategies in Saudi Arabia and Iraq could benefit educational practices across the region. These differences were attributed to the diverse educational practices and institutional support systems in place within each country, Table 4.

**Table 4:** Impact of sociodemographic characteristics on DREEM and TQ scores (*n* = 1246)

Socio-demographic characteristics	DREEM				TQ			
	Mean	±SD	Test	*p*	Mean	±SD	Test	*p*
Age (Yeas)								
< 17	103.04	35.67	**F =**		83.48	25.92	**F =**	
17–21	114.65	28.30	**2.225**	**0.109**	95.83	17.90	5.157*	0.006*
> 21	113.09	26.55			95.31	18.11		
Sex								
Male	109.97	29.36	**t =**		93.53	19.80	**t =**	
Female	116.61	26.19	4.200*	< 0.001*	96.75	16.85	3.015*	0.003*
Residence								
Urban	114.47	28.13	**t =**		95.63	18.51	**t =**	
Rural	111.81	26.62	**1.516**	**0.130**	94.66	17.34	**0.854**	**0.393**
Countries								
Egypt	97.04	11.63			90.29	15.21		
Iraq	119.34	26.40			96.62	17.61		
Saudi Arabia	126.79	27.26	**F =**		102.52	17.93	**F =**	
Yemen	117.96	25.47	27.746*	< 0.001*	95.97	18.29	10.389*	< 0.001*
Syria	108.96	33.72			91.82	19.47		
Income								
Good	116.40	30.77	**F =**		96.70	19.35	**F =**	
Fair	113.35	25.21	9.513*	< 0.001*	95.11	17.04	4.960*	0.007*
Poor	101.68	27.53			89.76	20.46		
Working during studying								
Not working	115.29	28.02	**F =**		96.07	18.15	**F =**	
Working and affecting my study	106.61	28.43	7.159*	0.001*	91.64	20.19	4.359*	0.013*
Working and not affecting my study	113.71	25.40			95.69	16.49		
Main reasons for working								
Earn more money	110.77	27.27	**t =**		93.63	17.78	**t =**	
More experience for may carrier	110.31	26.56	**0.164**	**0.870**	94.37	19.14	**0.387**	**0.699**
Financial support for students								
I don’t know	119.62	27.44	**F =**		96.92	19.40	**F =**	
No	109.83	27.09	17.588*	< 0.001*	93.68	17.74	3.779*	0.023*
Yes	110.70	27.81			95.60	16.80		
Faculty								
Nursing	119.91	26.23			97.19	18.59		
Medicine	107.41	29.95	**F =**		93.04	17.88	**F =**	
Pharmacy	108.60	21.10	12.207*	< 0.001*	94.95	15.65	3.338*	0.005*
Dentistry	107.15	26.08			95.23	18.38		
Technical health	117.92	28.03			98.51	16.16		
Physical therapy	110.74	26.87			90.85	21.34		
Academic year								
1st	117.83	32.43			92.80	24.35		
2nd	107.65	27.53	**F =**		93.41	18.00	**F =**	
3rd	111.36	26.47	12.398*	< 0.001*	93.50	16.52	7.968*	< 0.001*
4th	114.53	25.73			96.74	17.64		
5th	105.53	22.80			93.35	15.07		
6th	125.80	27.84			102.40	16.71		
Academic achievement								
Not trapped	115.98	27.58	**t =**		96.31	17.84	**t =**	
Trapped	101.07	25.37	6.833*	< 0.001*	89.99	19.47	4.107*	< 0.001*
Any support for trapped students								
Yes	120.59	28.09	**t =**		96.55	19.01	**t =**	
No	104.56	24.48	10.730*	< 0.001*	93.80	16.97	2.680*	0.007*
Lives with								
With Family	116.95	27.78	**t =**		96.95	17.84	**t =**	
Without family	104.55	25.61	7.292*	< 0.001*	90.80	18.57	5.148*	< 0.001*
Support center for students								
I don’t know	119.10	26.23	**F =**		97.14	17.97	**F =**	
No	104.15	26.50	34.579*	< 0.001*	91.66	18.01	10.542*	< 0.001*
Yes	113.36	29.73			95.99	18.46		

### Predictors of academic thriving

A multiple linear regression model was conducted to identify predictors of academic thriving. The total DREEM score, gender, family income, year of study, and awareness of support services were included as independent variables. The model was statistically significant accounting for 38.7% of the variance in thriving scores (adjusted *R*² = 0.387). Academic self-perception was the strongest predictor (β = 0.34, *p* < 0.001), followed by social self-perception (β = 0.28, *p* < 0.001) and awareness of support services (β = 0.17, *p* = 0.004), Table 5.

**Table 5:** Linear Regression Analysis of DREEM Dimensions on Student Outcomes (*n*= 1246)

DREEM	B	t	*p*	95% CI	
				LL	UL
Students’ Perception of Learning (SpoL)	0.135	1.648	0.100	−0.026	0.296
Students’ Perception of Teaching (SpoT)	0.092	1.128	0.260	−0.068	0.252
Students’ Academic Self-Perception (SASP)	1.330	13.974*	< 0.001*	1.144	1.517
Students’ Perception of the Atmosphere (SpoA)	0.119	1.479	0.140	−0.039	0.276
Students’ Social Self-Perception (SSSP)	0.571	4.652*	< 0.001*	0.330	0.812
R2 = 0.438, Adjusted R2 = 0.435, F = 193.083*, *p* < 0.001*					

R2: Coefficient of determination

B: Unstandardized Coefficients

*t* t-test of significance, *CI* Confidence interval, *LL* Lower limit, *UL* Upper Limit

*Statistically significant at *p* ≤ 0.05

The model explains 38.7% of the variance in student outcomes, as indicated by the *R*² value, suggesting that a substantial portion of the outcomes can be attributed to students’ perceptions of their educational environment. The 95% confidence interval (CI) ranges from 0.380 to 0.437, reinforcing the reliability of these findings. This analysis underscores the importance of fostering a positive educational atmosphere, as improvements in the DREEM score are likely to enhance overall student experiences and outcomes. The high F-value of 786.370 further confirms the robustness of the model, indicating that the predictors used are collectively significant in explaining the variance in student outcomes.

The regression analysis examines the impact of various dimensions of the DREEM on student outcomes, revealing significant findings for several key areas. Notably, Students’ Academic Self-Perception (SASP) shows a strong positive effect with an unstandardized coefficient (B) of 1.330, a t-value of 13.974, and a *p*-value of < 0.001, indicating a highly significant relationship. Similarly, Students’ Social Self-Perception (SSSP) also demonstrates a significant positive influence with a B of 0.571 and a *p*-value of < 0.001. Conversely, dimensions such as Students’ Perception of Learning (SpoL), Students’ Perception of Teaching (SpoT), and Students’ Perception of the Atmosphere (SpoA) do not show statistically significant effects, as indicated by their higher *p*-values. The overall model explains 43.8% of the variance in student outcomes (*R*² = 0.438), highlighting the considerable impact of academic and social self-perceptions on students’ experiences. The high F-value of 193.083 further reinforces the strength of the model, suggesting that while some dimensions are critical, others may require further investigation to understand their roles in shaping student outcomes.

## Discussion

This study investigated the relationship between the educational environment and academic thriving among medical students across five Arabic-speaking countries. The findings offer important insights into how specific elements of the educational climate can support or hinder student engagement, determination, and personal growth. Overall, the data reveals a generally positive perception of the learning environment, and a strong predictive relationship between certain DREEM domains—particularly academic and social self-perception—and thriving outcomes. While students generally perceived their learning environment as more positive than negative (mean DREEM = 113.79), there were notable variations in how different aspects of the environment influenced academic thriving. Consistent with similar studies conducted in medical schools within the MENA region [13–15], our results indicated that academic thriving was significantly associated with both academic self-perception and social self-perception. These findings highlight the importance of fostering confidence in students’ abilities and creating inclusive, supportive environments where students feel connected and valued. In comparison to our findings, Fleishman (2024) reveals their unique definitions of success and highlights the role of academic coaching in fostering a thriving environment. Ultimately, the findings suggest that students should play a central role in defining their own success, urging institutions to adopt thriving as a key element of success and to create supportive, student-centered cultures that align with individual goals and well-being.

Among the five subdomains, students reported the highest ratings for academic self-perception (SAP) and social self-perception (SSP), suggesting that their confidence in learning and their sense of peer support are key pillars of a supportive educational experience. These findings reinforce the significance of academic self-efficacy, a component strongly associated with thriving. As previously discussed by Schreiner in 2010, students who believe in their capacity to succeed academically are more likely to demonstrate cognitive engagement and resilience in the face of challenges [11, 12]. In parallel, social connectedness, reflected through high SSP scores, is aligned with models of student success that highlight the importance of belongingness and interpersonal support [16, 17]. The Thriving Quotient (TQ) subscales of engaged learning and academic determination were positively correlated with all DREEM subdomains, but strongest associations were found with SAP and SSP. These associations are not surprising, as engaged learners tend to draw motivation from self-efficacy and a meaningful academic identity [11]. A study by Edens and Froyen (2020) indicates that Academic Determination and Engaged Learning exert the greatest influence on student thriving.

Similarly, students who perceive social support within their learning environment are more likely to persist and thrive, especially in high-pressure disciplines such as medicine [18, 19]. The regression model further clarifies the predictive strength of these environmental components. Academic self-perception emerged as the most significant predictor of academic thriving (β = 0.34, *p* < 0.001), followed by social self-perception and awareness of support services. These findings are critical in contexts where institutional infrastructure may vary significantly across countries. While much literature emphasizes faculty support and curriculum design, this study suggests that internalized student beliefs and peer dynamics may have an even greater influence on academic flourishing.

Additionally, demographic variables such as gender, family income, and year of study were significant predictors of thriving. Female students reported higher mean scores in both DREEM and TQ compared to male students, which may be attributed to greater academic commitment or help-seeking behaviors, as reported in earlier studies [13, 18]. These findings suggest also that female students perceive their educational environment and the quality of teaching more positively, highlighting the need for educational institutions to consider gender-specific strategies to enhance academic experiences and support services for both male and female students. Students in the clinical years also demonstrated higher levels of thriving, potentially due to more immersive, purpose-driven learning experiences and greater alignment with professional identity development [19]. 6th-year students achieved the highest DREEM and TQ scores, suggesting a positive evolution in perceptions as students’ progress. A similar trend in female significance was observed in a study at King Abdulaziz University Faculty of Dentistry (KAUFD) in Saudi Arabia, which assessed dental students’ perceptions of their educational environment and its influence on academic achievement. Utilizing the DREEM questionnaire, the study gathered data from second-, third-, and fourth-year dental students. But conversely to our study results, third-year students reported higher scores than both fourth- and fifth-year students (Sabbagh et al., 2020).

Nursing students reported the highest DREEM score, indicating a favorable educational environment, while Pharmacy also scored significantly higher than Medicine and Dentistry. In terms of TQ scores, Nursing again led, with Pharmacy showing notable quality compared to Medicine and Dentistry. This suggests a particularly positive educational environment, which may be attributed to several factors. First, nursing curricula often emphasize hands-on training and mentorship, fostering a supportive learning atmosphere. Second, nursing programs frequently incorporate teamwork and collaboration, enhancing peer relationships and engagement. Third, the emphasis on patient-centered care in nursing education may lead to greater student motivation and satisfaction. Fourth, faculty members in nursing may prioritize student feedback and adapt teaching methods accordingly, improving overall teaching quality. Lastly, the strong sense of community within nursing programs can contribute to a more cohesive and encouraging educational experience, allowing students to thrive compared to their peers in Pharmacy, Medicine, and Dentistry. Aligning with findings from Farooq et al. (2018) in Pakistan, who found that nursing students had a more favorable perception of their educational environment compared to medical students.

Interestingly, those who are Working and not affecting their study have DREEM scores and TQ scores (95.69) that are closer to the non-working group, indicating that if students can manage their work commitments effectively without detrimental effects on their studies, they may still maintain a positive educational experience. The significant F-statistics (7.159 for DREEM and 4.359 for TQ) and the corresponding *p*-values (0.001 and 0.013, respectively) indicate strong evidence that working status is an important factor influencing both academic thriving and perceived teaching quality among medical students. These findings underscore the necessity for educational institutions to consider the work-life balance of students. Implementing support systems, such as flexible scheduling or resources for time management, could help mitigate the negative effects of work on academic thriving. A recent longitudinal study by Dekker (2022) investigated the factors that contribute to academic thriving among students. The findings revealed that students with paid jobs in education earned more study credits compared to those with other types of employment or those who were unemployed.

Notably, domains such as perception of atmosphere (SPA) and perception of teachers (SPT), while still positively correlated with thriving, exhibited relatively weaker predictive power. This may indicate that although a conducive institutional climate and faculty-student interactions are important, they do not directly influence thriving unless mediated by students’ internal perceptions and relationships. The observed differences in educational environment and thriving across Middle Eastern countries can be attributed to several interrelated factors. First, investment in education plays a crucial role in shaping outcomes. Countries such as Saudi Arabia and Iraq have made significant financial commitments to enhance their educational systems, focusing on infrastructure, resources, and teacher training. In contrast, Egypt and Syria face budget constraints that hinder their ability to improve educational facilities and support, which is reflected in their lower DREEM scores. Cultural and societal factors also contribute to these disparities. In nations where there is a strong societal emphasis on education, community support tends to foster better educational environments. Saudi Arabia exemplifies this cultural commitment, while Egypt and Syria may struggle due to societal challenges and conflicts that detract from community engagement with educational institutions. This lack of support can adversely affect student experiences and perceptions of educational quality. Political stability emerges as another critical determinant. Countries like Saudi Arabia, with relative political stability, can implement consistent educational policies and reforms effectively.

On the other hand, Egypt and Syria’s political unrest disrupts educational processes and hinders the execution of reforms, leading to inconsistent educational quality and environments. This instability further complicates efforts to enhance teaching and learning experiences. Teacher quality and training are fundamental to educational success. The prioritization of teacher recruitment and professional development in Saudi Arabia and Iraq has resulted in better-prepared educators, which positively impacts student learning. Conversely, Egypt and Syria may experience challenges such as teacher shortages and inadequate training, leading to less effective teaching practices and lower perceptions of teaching quality.

Finally, access to resources and technology significantly influences educational experiences. Saudi Arabia’s better access to modern educational tools and materials enhances the learning environment, whereas Egypt and Syria may face resource shortages that limit students’ exposure to diverse learning opportunities. This lack of resources not only affects educational outcomes but also contributes to the lower scores in both DREEM and TQ assessments in these countries. The study by Valdois (2019) highlights the critical role of culture in determining student thriving, particularly among Chinese students in an American program at a private university in Henan province, China. It argues that traditional success metrics like grades and graduation rates fail to capture the complexities of student experiences. Utilizing Schreiner’s thriving model, the research examines factors such as Engaged Learning, Academic Determination, and Psychological Sense of Community (PSC), which is particularly significant in China’s collective society. While PSC can enhance thriving, the cross-cultural environment may challenge students’ sense of community.

Future research may explore whether faculty development programs that promote relational pedagogy can indirectly enhance students’ sense of autonomy and belonging. These findings carry practical implications. First, student support initiatives, including academic advising, peer mentoring, and wellness programs—should be reinforced as they directly contribute to thriving. Second, curricular and assessment strategies should be evaluated to ensure they foster mastery experiences that build students’ academic self-efficacy. Third, medical schools must recognize that student success is not solely academic, but also psychosocial, and that interventions should reflect this holistic view. This study also adds value by using a multinational and multicenter design, offering a broader perspective that surpasses the limitations of single-institution studies. The diversity of the sample strengthens the generalizability of findings within the Arab region, where sociocultural and institutional variability can significantly influence medical education outcomes.

Despite its strengths, this study has several limitations. The cross-sectional design limits causal inference, and self-reported data may be subject to social desirability or recall bias. The use of an online survey may have excluded students with limited internet access or lower engagement levels. Moreover, although the sample was diverse, it may not represent all medical schools or sociocultural contexts within the region.

Future studies may benefit from using longitudinal designs to track thriving over time and from incorporating qualitative methods to gain deeper insights into how students perceive and navigate their educational environments. Further validation of the Thriving Quotient in Arabic-speaking populations is also recommended.

## Conclusions

This study represents a significant advancement in educational research as the first multinational Arabic study conducted in the MENA region, examining the interplay between educational environments and academic thriving among medical students across multiple Arabic-speaking countries. It provides valuable insights into the unique challenges and opportunities faced by these students. The findings highlight the importance of contextual factors in shaping academic experiences and underscore the need for tailored interventions that acknowledge the cultural and institutional diversity within the region. By emphasizing the roles of academic self-perception and social support, this research reveals critical predictors of student engagement and personal growth. The observed variations across demographic groups further illustrate the necessity for strategies that consider cultural, political, and societal contexts. As educational institutions aim to enhance student success, a holistic approach integrating both academic and psychosocial support systems is essential. Future research should continue to explore these dimensions, employing diverse methodologies to capture the complexities of student experiences in medical education.

## Supplementary Information

Supplementary Material 1.

## Data Availability

The datasets used and/or analysed during the current study are available from the corresponding author on reasonable request.

## Abbreviations

DREEM: Dundee Ready Education Environment Measure
TQ: Thriving Quotient
SPSS: Statistical Package for the Social Sciences
SAP: Students’ Academic Self-Perception
SSP: Students’ Social Self-Perception
SPT: Students’ Perception of Teaching
SPL: Students’ Perception of Learning
SPA: Students’ Perception of the Atmosphere
CI: Confidence Interval
R²: Coefficient of Determination
