# Supplementary material for: The impact of educational environment on academic thriving among medical students: insights from a multinational cross-sectional survey
Source: BMC Med Educ. 2025 Oct 21;25:1449. doi: 10.1186/s12909-025-08053-2 (PMC12538949; doi:10.1186/s12909-025-08053-2)
Supplement: Supplementary file 1 — Supplementary Material 1. [file 12909_2025_8053_MOESM1_ESM.docx]

**The Impact of Educational Environment on Academic Thriving among Medical Students: Insights from a Multinational Cross-sectional Survey**

**Informed Consent Form**

I agree to participate in the research voluntarily, and I understand that I have the freedom to withdraw from the research at any time without any negative consequences. Information and data will be collected via social media, WhatsApp groups, or personal interviews and will be used solely for research purposes. Once you complete the questionnaire, you agree to participate in the research.

**Dear student,** here is the questionnaire for a study to explore the relationship between the learning environment and academic success among medical students: Insights from a multinational study. Your individual privacy and the confidentiality of the information you provide will be maintained throughout the analysis of the written data resulting from the study.

Your participation in this survey will help improve teaching and assessment strategies in medical schools, contributing to the development of students' skills and increasing their competency in healthcare.

---------------------------------------------------------------------------------------------------------------------

**Tool I: Student Demographic characteristics, Academic status, Health, Social, Psychological and Economic characteristics Interview Schedule**

1. Age

| Under 17 years old ( ) | From 18 to 20 years old ( ) | Over 21 years old ( ) |
| --- | --- | --- |

1. Sex

| Male ( ) | Female ( ) |
| --- | --- |

1. Residence

| Rural ( ) | Urban ( |
| --- | --- |

1. Evaluate my economic situation:

| Good ( ) | Moderate ( ) | Poor ( ) |
| --- | --- | --- |

1. Working while studying

| I do not work ( ) | work and the work affects my academic achievement ( ) | I work and the work does not affect my academic achievement ( ) |
| --- | --- | --- |

1. Main reason for working:

| To fulfill my personal and family needs ( ) | To improve my practical experience in my field ( ) |
| --- | --- |

1. The college provides a financial and economic support center for students:

| I don't know ( ) | Not available ( ) | Available ( ) |
| --- | --- | --- |

1. College:

| Nursing | Medicine | Pharmacy |
| --- | --- | --- |
| Dentistry | Health Technologies | Physical Therapy |

1. Current academic year:

| First | Second | Third |
| --- | --- | --- |
| Fourth | Fifth | Sixth |

1. Study status:

| I am a non-disrupting student, regular with my academic level ( ) | I am a discontinuing student, irregular with my academic level ( ) |
| --- | --- |

1. Academic support is provided to struggling students:

| Yes ( ) | No ( ) |
| --- | --- |

1. I live:

| Within the family ( ) | Outside the family ( ) |
| --- | --- |

1. Providing a social support and case study center for students at the college:

| I don't know ( ) | Not available ( ) | Available ( ) |
| --- | --- | --- |

1. Health status:

| I have health problems ( ) | I do not have health problems ( ) |
| --- | --- |

1. Take medication regularly:

| Yes ( ) | No ( ) |
| --- | --- |

1. You are suffering from serious health conditions that require immediate medical intervention:

| Yes ( ) | No ( ) |
| --- | --- |

1. Providing a health support center for students at the college:

| I don't know ( ) | Not available ( ) | Available ( ) |
| --- | --- | --- |

1. Psychological state:

| I suffer from a mental illness or psychological problems ( ) | I do not suffer from a mental illness or psychological problems |
| --- | --- |

1. The college provides a psychological support center for students:

| I don't know ( ) | Not available ( ) | Available ( ) |
| --- | --- | --- |

1. Interests, hobbies, and artistic/sporting/cultural practices:

| Yes, I have one of them ( ) | I don't have any of them ( ) |
| --- | --- |

1. Participation in student activities:

| I like to participate in student activities at the college or university level ( ) | I do not like to participate in student activities at the college or university level ( ) |
| --- | --- |

1. Providing a support, care and development center for student activities for students at the college:

| I don't know ( ) | Not available ( ) | Available ( ) |
| --- | --- | --- |

**Tool II: Dundee Ready Educational Environment Measure (DREEM)**

| Items | strongly agree | agree | unsure | disagree | strongly disagree |
| --- | --- | --- | --- | --- | --- |
| **First domain: Students’ Perception of Learning (SpoL** | | | | | |
| 1- I am encouraged to participate in teaching sessions |  |  |  |  |  |
| 2- The teaching is often stimulating |  |  |  |  |  |
| 3- The teaching is student-centered |  |  |  |  |  |
| 4- The teaching helps to develop my competence |  |  |  |  |  |
| 5- The teaching is well-focused |  |  |  |  |  |
| 6- The teaching helps to develop my confidence |  |  |  |  |  |
| 7-The teaching time is put to good use |  |  |  |  |  |
| 8-The teaching over-emphasizes factual learning |  |  |  |  |  |
| 9-I’m clear about the learning objectives of the course |  |  |  |  |  |
| 10-The teaching encourages me to be an active learner |  |  |  |  |  |
| 11- Long-term learning is emphasized over short-term learning |  |  |  |  |  |
| 12- The teaching is too teacher-centered |  |  |  |  |  |
| **Second domain: Students’ Perception of Teachers (SPoT)** | | | | | |
| 1-The teachers are knowledgeable |  |  |  |  |  |
| 2-The teachers adopt a patient-centred approach to consulting |  |  |  |  |  |
| 3-The teachers ridicule the students |  |  |  |  |  |
| 4- The teachers are authoritarian |  |  |  |  |  |
| 5- The teachers have good communication skills with patients |  |  |  |  |  |
| 6-The teachers are good at providing feedback to students |  |  |  |  |  |
| 7-The teachers provide constructive criticism here |  |  |  |  |  |
| 8- The teachers give clear examples |  |  |  |  |  |
| 9- The teachers get angry in teaching |  |  |  |  |  |
| 10-The teachers are well-prepared for their teaching sessions |  |  |  |  |  |
| 11- The students irritate the teachers |  |  |  |  |  |
| **Third domain ; Students’ Academic Self-Perception (SASP)** | | | | | |
| 1-Learning strategies that worked for me before continue to work for me now |  |  |  |  |  |
| 2- I am confident about my passing this year |  |  |  |  |  |
| 3- I fell I am being well prepared for my profession |  |  |  |  |  |
| 4- Last year’s work has been a good preparation for this year’s work |  |  |  |  |  |
| 5- I am able to memorize all I need |  |  |  |  |  |
| 6- I have learnt a lot about empathy in my profession |  |  |  |  |  |
| 7-My problem-solving skills are being well developed here |  |  |  |  |  |
| 8- Much of what I have to learn seems relevant to a career in healthcare |  |  |  |  |  |
| **Fourth domain ; Students’ Perception of Atmosphere (SPoA)** | | | | | |
| 1- The atmosphere is relaxed during ward teaching |  |  |  |  |  |
| 2-This school is well time-tabled |  |  |  |  |  |
| 3-Cheating is a problem in this school |  |  |  |  |  |
| 4-The atmosphere is relaxed during lectures |  |  |  |  |  |
| 5-There are opportunities for me to develop my interpersonal skills |  |  |  |  |  |
| 6-I feel comfortable in class socially |  |  |  |  |  |
| 7-The atmosphere is relaxed during class/ seminars/tutorials |  |  |  |  |  |
| 8-I find the experience disappointing |  |  |  |  |  |
| 9-I am able to concentrate well |  |  |  |  |  |
| 10-The enjoyment outweighs the stress of the course |  |  |  |  |  |
| 11- The atmosphere motivates me as a learne |  |  |  |  |  |
| 12- I feel able to ask the questions I want |  |  |  |  |  |
| **Fifth domain ; Students’ Social Self-Perception (SSSP)** | | | | | |
| 1-There is a good support system for students who get stressed |  |  |  |  |  |
| 2- I am too tired to enjoy the course |  |  |  |  |  |
| 3- I am rarely bored in this course |  |  |  |  |  |
| 4-I have good friends in this course |  |  |  |  |  |
| 5- My social life is good |  |  |  |  |  |
| 6-I seldom feel lonely |  |  |  |  |  |
| 7- My accommodation is pleasant |  |  |  |  |  |

**Tool III: Thriving Quotient Survey Instrument (25-item) (version 2020-2021)**

| **1** | **2** | **3** | **4** | **5** | **6** |
| --- | --- | --- | --- | --- | --- |
| ***very dissatisfied*** | ***dissatisfied*** | ***somewhat dissatisfied*** | ***somewhat satisfied*** | ***satisfied*** | ***very satisfied*** |

| **Engaged Learning (ELI) (four items** |
| --- |
| 1-I feel as though I am learning things in my classes that are worthwhile to me as a person |
| 2-I can usually find ways of applying what I’m learning in class to something else in my life |
| 3-I find myself thinking about what I’m learning in class even when I'm not in class |
| 4-I feel energized by the ideas I am learning in most of my classes |
| **Academic Determination (AD) (six items)** |
| 5-I am confident I will reach my educational goals |
| 6-Even if assignments are not interesting to me, I find a way to keep working at them until they are done well |
| 7-I know how to apply my strengths to achieve academic success |
| 8-I am good at juggling all the demands of college life |
| 9-Other people would say I’m a hard worker |
| 10-When I’m faced with a problem in my life, I can usually think of several ways to solve it. |
| 11-THIS WEEK, When I’m faced with a problem in my life, I can usually think of several ways to solve it. |
| **Diverse Citizenship (DC) (six items)** |
| 12-I spend time making a difference in other people’s lives |
| 13-I know I can make a difference in my community |
| 14-I value interacting with people whose viewpoints are different from my own |
| 15-It’s important for me to make a contribution to my community |
| 16-It is important to become aware of the perspectives of individuals from different backgrounds |
| 17-My knowledge or opinions have been influenced or changed by becoming more aware of the perspectives of individuals from different backgrounds. |
| **Positive Perspective (POS) (two items)** |
| 18-My perspective on life is that I tend to see the glass as ‘half full’ rather than ‘half empty’ |
| 19-I look for the best in situations, even when things seem hopeless. |
| **Social Connectedness (SC) (Six items)** |
| 20-Other people seem to make friends more easily than I do **(reverse score)** |
| 21-I feel like my friends really care about me |
| 22-I don’t have as many close friends as I wish I had **(reverse score)** |
| 23-I feel content with the kinds of friendships I currently have |
| 24-I often feel lonely because I have few close friends with whom to share my concerns **(reverse score)** |
| 25-It’s hard to make friends at this institution **(reverse score)** |

**Good Luck**
